# A time course-dependent metastatic gene expression signature predicts outcome in human metastatic melanomas

**DOI:** 10.1186/s13000-014-0155-2

**Published:** 2014-08-13

**Authors:** Rongyi Chen, Guoxue Zhang, Ying Zhou, Nan Li, Jiaxi Lin

**Affiliations:** Department of Dermatology, Affiliated Hospital of Guangdong Medical College, Zhanjiang, 524001 China

**Keywords:** Melanomas, Metastasis, Prognosis, Prediction, Gene signature

## Abstract

**Background:**

The prognosis of patients with metastatic melanomas is extremely heterogeneous. Therefore, identifying high-risk subgroups by using innovative prediction models would help to improve selection of appropriate management options.

**Methods:**

In this study, two datasets (GSE7929 and GSE7956) of mRNA expression microarray in an animal melanoma model were normalized by frozen Robust Multi-Array Analysis and then combined by the distance-weighted discrimination method to identify time course-dependent metastasis-related gene signatures by Biometric Research Branch-ArrayTools (BRB)-ArrayTools. Then two datasets (GSE8401 and GSE19234) of clinical melanoma samples with relevant clinical and survival data were used to validate the prognosis signature.

**Results:**

A novel 192-gene set that varies significantly in parallel with the increasing of metastatic potentials was identified in the animal melanoma model. Further, this gene signature was validated to correlate with poor prognosis of human metastatic melanomas but not of primary melanomas in two independent datasets. Furthermore, multivariate Cox proportional hazards regression analyses demonstrated that the prognostic value of the 192-gene set is independent of the TNM stage and has higher areas under the receiver operating characteristic curve than stage information in both validation datasets.

**Conclusion:**

Our findings suggest that a time course-dependent metastasis-related gene expression signature is useful in predicting survival of malignant melanomas and might be useful in informing treatment decisions for these patients.

**Electronic supplementary material:**

The online version of this article (doi:10.1186/s13000-014-0155-2) contains supplementary material, which is available to authorized users.

## Background

The incidence of malignant melanoma, an aggressive form of skin cancer, is increasing rapidly globally, including in China [1]. It has been estimated that approximately 15% of patients with primary melanoma will develop distant metastases [2]. Because metastatic melanoma responds poorly to conventional therapy, its outcome is generally very poor [3,4]. However, a proportion of patients do survive for prolonged periods following development of metastasis. Therefore, developing innovative prediction models for stratifying metastatic melanoma further would improve planning of appropriate managements.

Gene expression profiling has been used to establish molecular signatures for classifying the subtypes of primary tumors and predicting the clinical outcome of multiple cancers including malignant melanoma [5,6]. Previous studies have identified several panels of gene expression signatures with clinical relevance to malignant melanoma. In this study, we identified a novel gene signature based on time course mRNA expression microarray data in an animal melanoma model that can be used to predict survival in patients with malignant melanomas.

## Methods

### Datasets

The gene expression profiles of four independent datasets deposited in the Gene Expression Omnibus (GEO) (accession numbers GSE7929, GSE7956, GSE8401, and GSE19234) were analyzed [7,8]. Microarray platforms used in these datasets were HU133A Affymetrix DNA chips (GSE7929, GSE7956, GSE8401) and Illumina HumanWG-6 v2 E/HumanWG HT12 Expression Beadchip (GSE19234).

The GSE7929 comprises the gene expression microarray data of 32 human melanoma cells samples including 11 replicas of parental melanoma cell line A375 (LeiATCC) and 21 derived cell lines with different metastatic potentials selected based on a xenotransplant metastasis model [7]. The GSE7956 dataset comprises whole-genome mRNA expression data of 39 human melanoma cell samples, including 10 replicas of parental melanoma cell line A375 (LeiFidler) and 29 derived cell lines with different metastatic potentials generated by two or three rounds of selection in immunodeficient mice [7].

The GSE8401 dataset was generated from 83 melanoma samples (31 primary and 52 metastatic tumors) that were collected from patients undergoing surgery were collected from 1992 to 2001 at the Massachusetts General Hospital and Harvard Medical School as a part of the diagnostic workup or planning of therapy [7]. Fifty-two of these metastatic melanoma samples were of superficially spreading type, 19 nodular, and 5 acral lentiginous; the types are not available for the remaining. Most of the primary melanomas were TNM stage I-II, the metastatic melanoma group includes 17 stage III and 35 stage IV tumors [7].

The GSE19234 dataset comprises 44 melanoma tumors with metastasis were collected at the New York University Medical Center from 1988 to 1997 [8]. Five cases received immunotherapy. Fifteen cases showed CD3 positive. Among these tumors, 4 cases were at stage IIIA tumors,23 at stage IIIB, 12 at stage IIIC, and 5 at stage IV [8].

As claimed in the original publications, all the studies on these datasets had been approved by the local ethical committees and samples were collected with written informed consent from the patients.

### Microarray data merging

Two human melanoma cell line datasets GSE7929 and GSE7956 were used to identifying time course-dependent metastasis-related gene signatures. All gene expression profiles were normalized with frozen Robust Multi-Array Analysis (fRMA), a procedure in which allows arrays are not longer clustered per study but per platform [9]. The two datasets were combined using the distance-weighted discrimination (DWD) method [10]. DWD is a multivariate analysis tool that is able to adjust experiments batch effects to compensate for biases present in different gene expression datasets. The integration effect was inspected by a box-plot of gene expression in each array using Biometric Research Branch (BRB)-ArrayTools 4.3.1 [11].

### Time course analysis of metastasis-related gene signature on merged dataset

A time course analysis of the combined datasets of GSE7929 and GSE7956 was performed with the expression profiles of different generations of metastatic cell lines set as time-course data. A BRB-ArrayTool plug-in was used to perform regression analysis of time course expression data to identify the genes whose expression varies over time. The round numbers of different generations of selected cells were set as different time points. The tests were performed at a false discovery rate (FDR) threshold of 0.05. A list of genes that satisfied this threshold was produced and used as gene sets to assess correlations with poor survival of patients with melanomas in the validation datasets.

### Validation of the time course prediction gene signature in independent datasets

Two clinical melanoma sample datasets (GSE8401 and GSE19234) were used to validate the prognostic significance of the time course-dependent metastasis-related gene signature. For the validated datasets, series matrix files containing normalized data were obtained from the GEO. Survival risk prediction of the gene expression data by the time course-dependent metastatic gene signature was performed based on principal components with BRB-ArrayTools software. Survival risk prediction was based on 10-fold cross-validation and classified patients into low- and high-risk groups according to the prognostic indexes.

### Statistics

Distributions of overall survival were assessed using the Kaplan-Meier curve method and log-rank statistics based on 100 permutations. Multivariate analyses of prognostic factors were based on the Cox proportional hazards model. Area under the receiver operating characteristic (ROC) curves were used as a performance measure for prognostic factors. Permutated Kaplan-Meier curve analysis was performed using BRB-ArrayTools. All other statistical analyses were performed with Medcalc Software. P values less than 0.05 were considered statistically significant.

## Results

**Figure 1:**
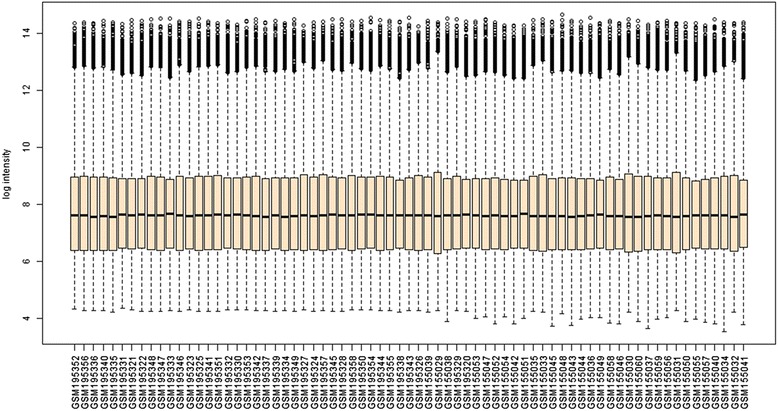
**A Box-plot of gene expression in each metastatic melanoma array of the combined dataset (GSE7929 and GSE7956).** Using distance-weighted discrimination (DWD) method after frozen Robust Multi-Array Analysis (fRMA) normalization indicates a good batch effect removal.

In this study, a gene set that correlated with the metastatic potential of melanoma cell lines was first identified from the gene expression profiling of two datasets (GSE7929 and GSE7956). To increase power, these two data sets were merged by the DWD method after fRMA normalization. As seen in Figure 1, after merging, the relative log gene expression plots indicated a good batch effect removal.

**Figure 2:**
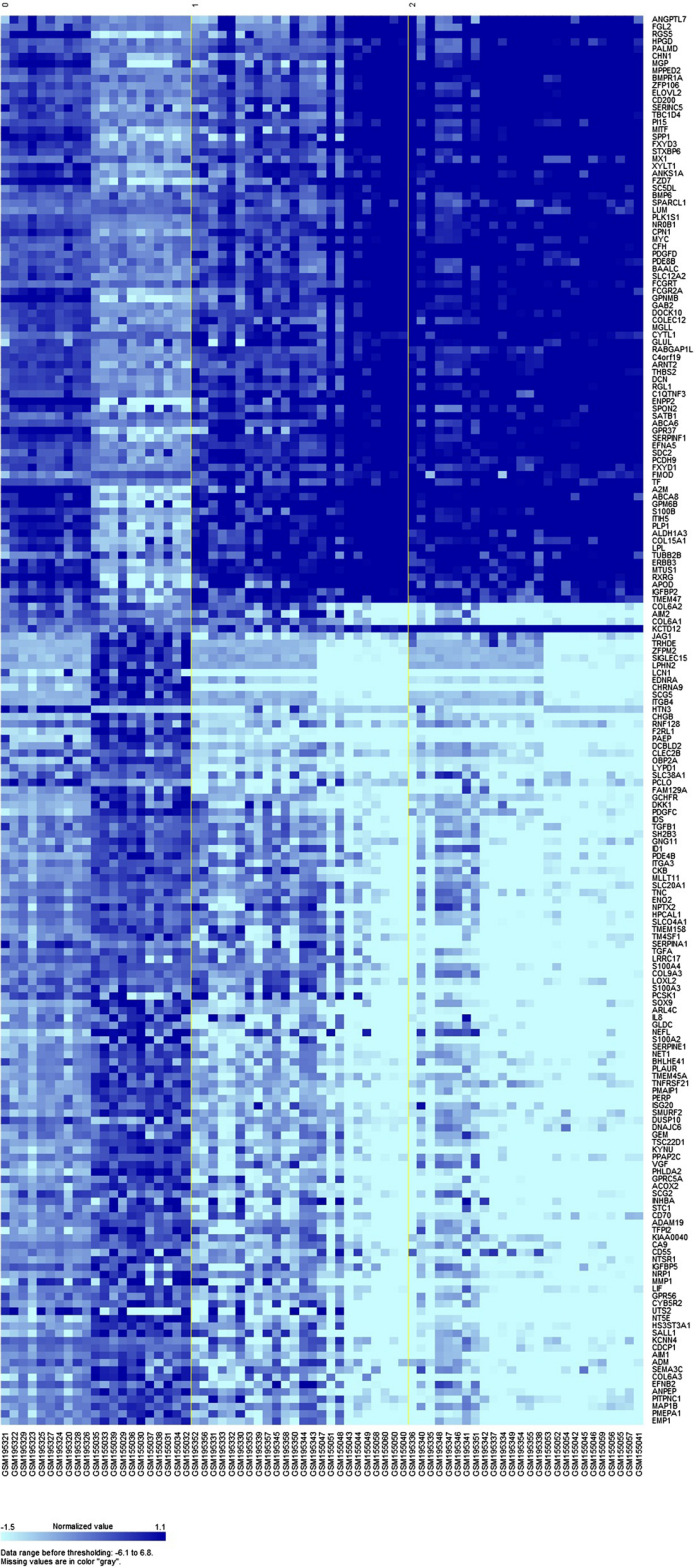
**Hierarchical clustering of samples of the combined datasets (GSE7929 and GSE7956) by the time course gene set.** Parental lines samples were assigned as “time course 0”; metastatic derived cell lines established after one round of selection as “time course 1” (n = 24); derived cell lines after two or three rounds of selection as “time course 2”.

The merged dataset comprised of 71 samples among which the parental lines samples were assigned as “time course 0” (n = 21); metastasis-derived cell lines established after one round of selection as “time course 1” (n = 24); derived cell lines after two or three rounds of selection as “time course 2” (n = 26). Cells at different metastatic stages had distinct expression patterns. As seen in Figure 2 and in the Additional file 1, 192 genes were identified by BRB-ArrayTools as changing significantly over the time course with an FDR threshold of 0.05. Furthermore, this 192-gene signature was compared with a previously identified 150-gene signature in GSE8401 and 266-gene signature in GSE19234. There was little gene overlap among these three gene sets, which indicates that our 192-gene signature is novel for predicting survival of patients with metastatic melanoma.

**Figure 3:**
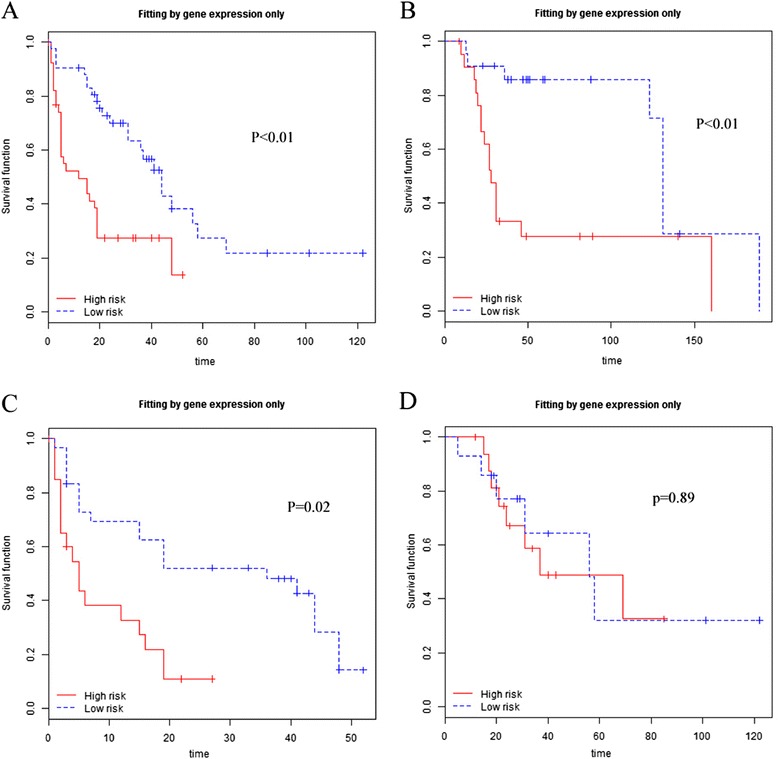
**Survival prediction using a 192-gene signature in two validation datasets.** High-risk group defined by a 192 gene set had a significantly shorter overall survival time than low-risk group in GSE8401 **(A)**, GSE19234 **(B)**, and metastatic tumor subgroup of GSE8401 **(C)**, but not in primary tumor subgroup of GSE8401 **(D)**. (By Kaplan-Meier curve method and log-rank statistics based on 100 permutations).

**Figure 4:**
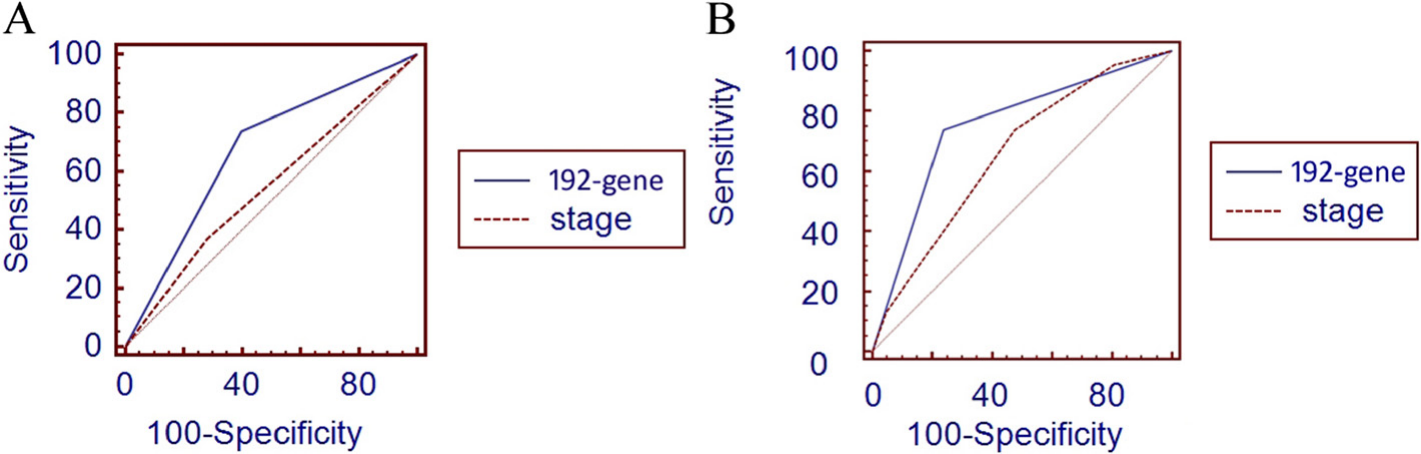
**Comparison of ROC curves between 192-gene set signature and TNM stage. A** 192-gene signature gave a predictive performance with higher areas under the ROC curve than those with stage information in both two validation datasets of metastatic melanoma (**A**: GSE840; **B**: GSE19234).

Next, the robustness with which the 192 metastasis-related gene expression signature in predicts survival in patients with melanomas was further tested with whole-genome mRNA expression profiling data obtained from two completely independent datasets (GSE8401 and GSE19234). Using the prognostic indexes based on our gene signature, patients were partitioned into high- and low-risk groups. As seen in Figure 3 the high-risk groups was observed to have a significantly shorter overall survival time than the low-risk group in 55 metastatic melanomas of GSE8401, and 44 metastatic melanomas of GSE19234. However, no significant difference in survival between high- and low-risk groups was observed for primary melanoma samples of GSE8401 Figure 4.

Multivariate Cox proportional hazards regression analyses showed that the prognostic value of our 192-gene signature was independent of TNM stage in both validation datasets (Table 1). The Cox model with the 192-gene signature was also found to provide a predictive performance with higher areas under the ROC curve than TNM stage.

**Table 1:** **Multivariate Cox proportional hazards regression analyses on survival time in two metastatic melanoma datasets**

**Covariate**	**P**	**Exp(b)**	**95% CI of Exp (b)**
GSE8401			
192-gene signature	0.0043	2.8517	1.3948 to 5.8301
Stage	0.0585	2.1791	0.9764 to 4.8633
GSE19234			
192-gene signature	0.0353	2.7182	1.0762 to 6.8655
Stage	0.0172	1.9844	1.1323 to 3.4778

## Discussion

Because metastatic melanomas are heterogeneous, it is critical to develop reliable clinical or molecular predictors to identify those with a relatively worse prognosis and thus select those that could benefit from more aggressive therapies. Previous studies have identified two survival-related gene signatures for patients with metastatic melanoma. The first signature, a 266-gene signature, was generated using the Significant Analysis of Microarrays (SAM) method and could enhance prediction of survival of metastatic melanoma patients by clinical staging [8]. A more earlier study by Xu et al. [7] compared the gene expression patterns of highly metastatic human melanoma cell lines derived from poorly metastatic parental lines in an animal metastasis model using the Gene Set Enrichment Analysis (GSEA) method and thus identified another survival-related 150-gene set for predicting prognosis of metastatic melanomas.

In this study, we re-analyzed the same dataset of animal melanoma models as that assessed by Xu et al. [7]. Unlike the original study, we used a time-course strategy to isolate metastasis-related genes. We identified 192 genes that varied significantly in parallel with increasing metastatic potential; this set differs from the previously identified gene sets. We confirmed that this time-course gene expression changes in an animal melanoma model correlate with survival of patients with melanoma metastases in two independent datasets. Moreover, our novel time-course gene signature predictor is independent of stage, and has better predictive performance than stage. Our findings suggest that this 192-gene signature might improve the ability of TNM staging to predict survival of patients with metastatic melanoma. Further, we also found that gene expression signatures from animal melanoma model and human melanoma datasets could be reconciled.

Interpretation of gene function showed that the 192 genes are enriched significantly in extracellular matrix receptor interaction, focal adhesion and the β-transforming growth factor signaling pathway. Many of these genes have previously been implicated in melanoma metastasis and prognosis prediction, including MYC, GPNMB, GAB2, and SATB1 [12-15]. Therefore, the functional significance of this 192-gene pathway also warrants further investigation.

## Conclusions

In conclusion, the time course-dependent gene signature we have developed is useful for predicting survival of patients with metastatic melanomas and might be useful for informing treatment decisions.

## Additional file

Additional file 1:
**A 192-gene set that varies significantly in parallel with the increasing of metastatic potentials of melanoma.**

## Abbreviations

DWD: Distance-weighted discrimination
fRMA: Frozen robust multi-array analysis
ROC: Receiver operating characteristic
SAM: Significant analysis of microarrays
GSEA: Gene set enrichment analysis
